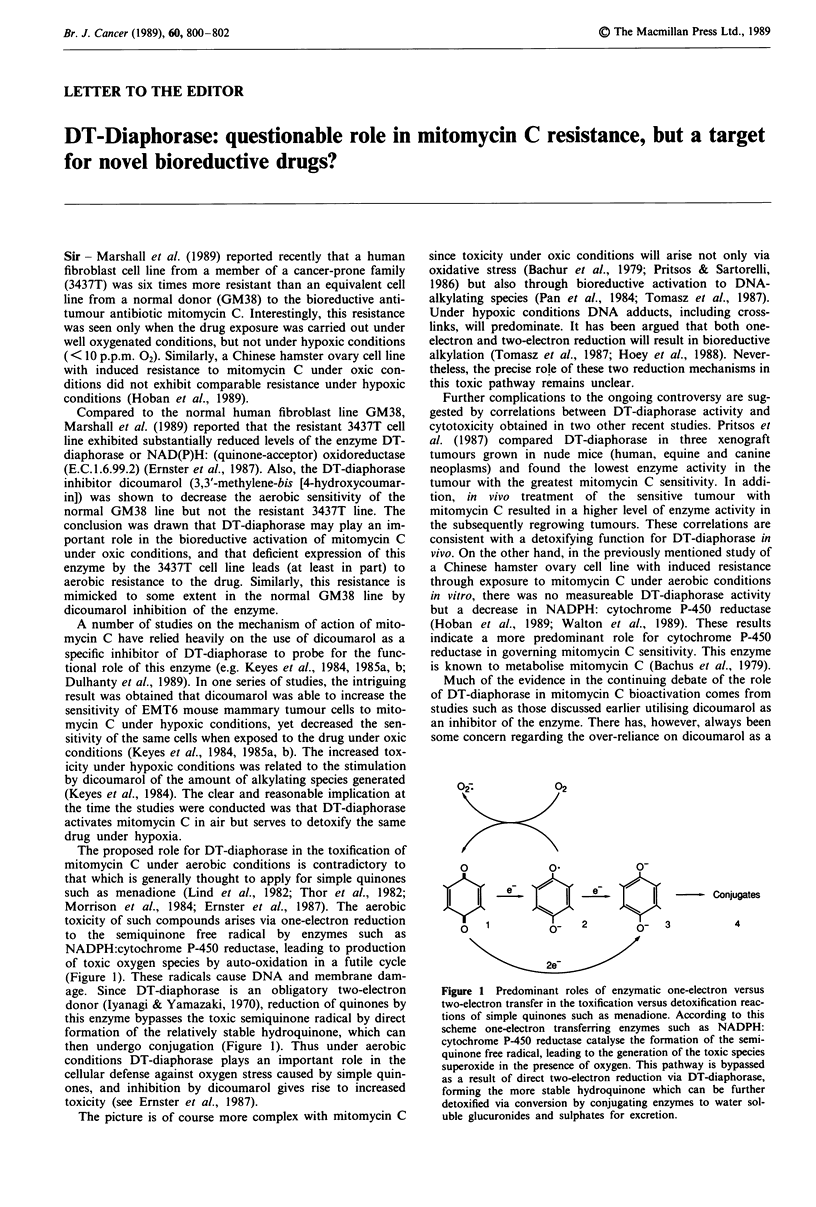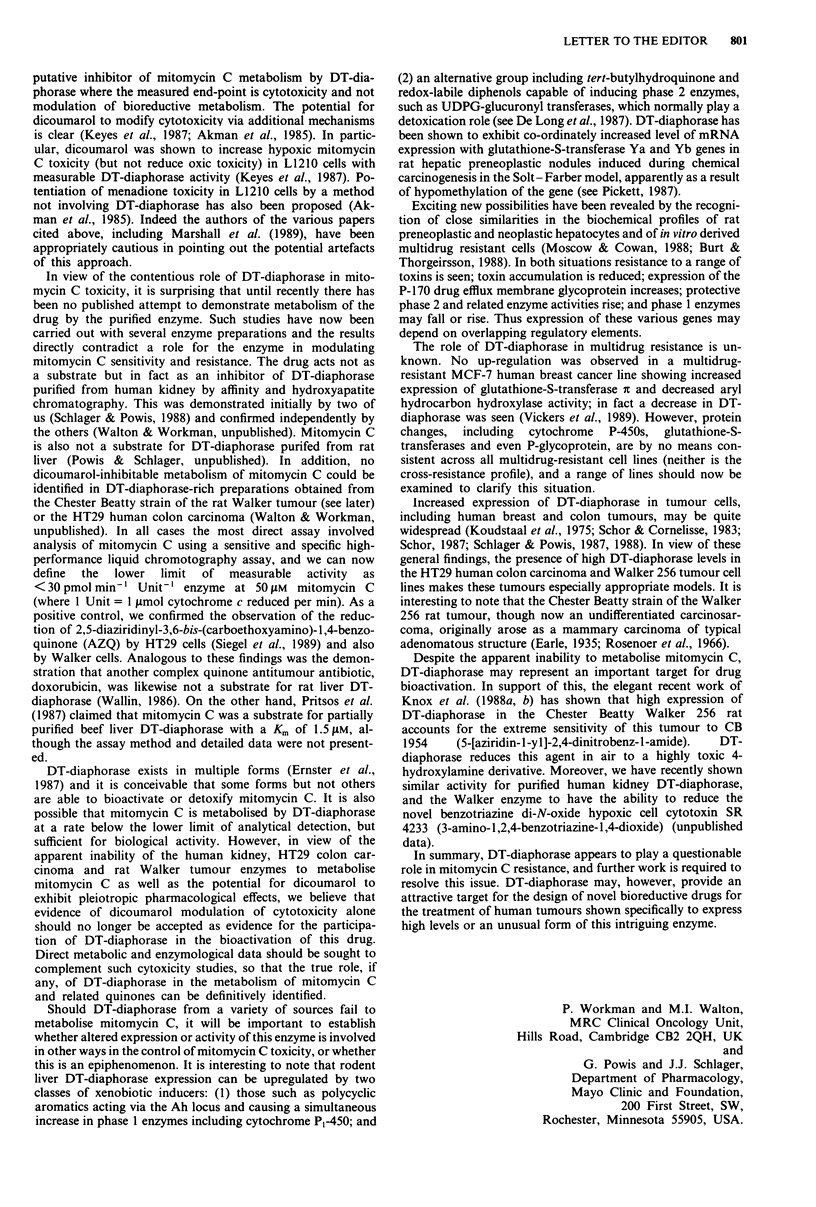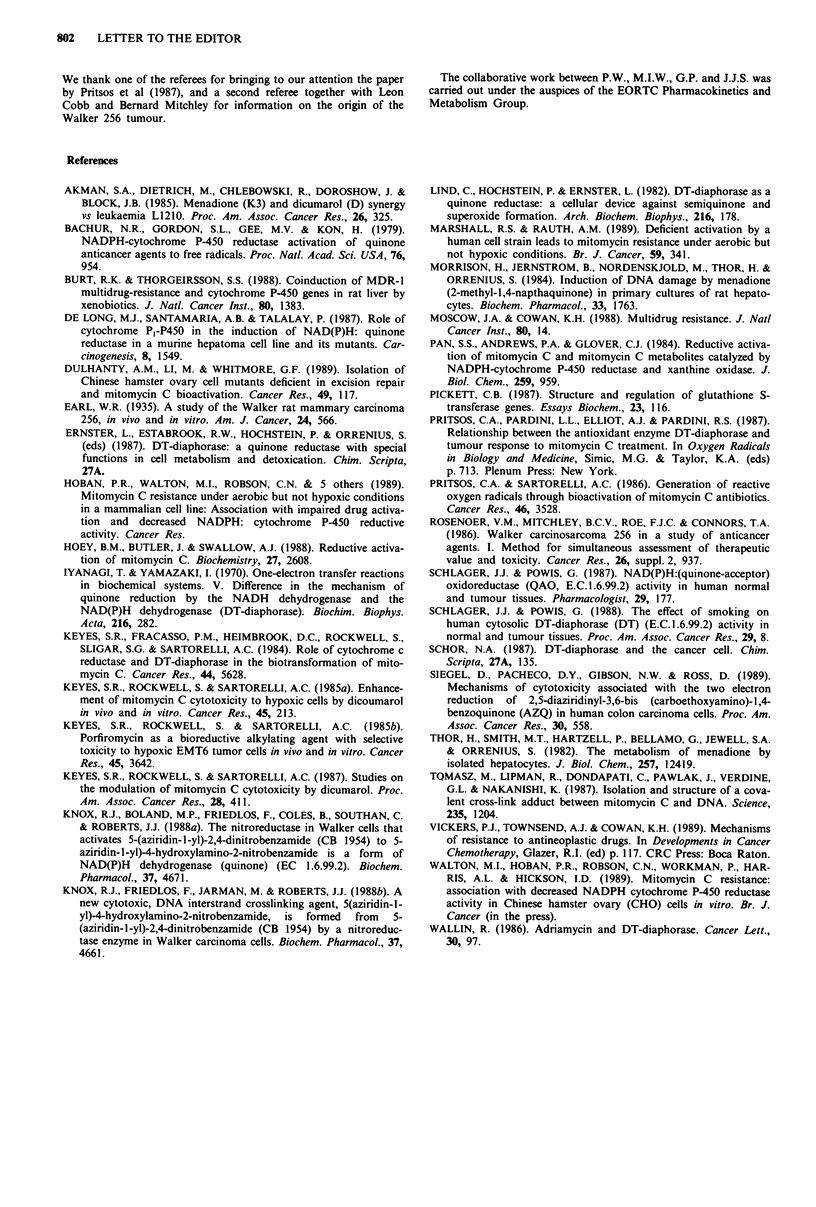# DT-diaphorase: questionable role in mitomycin C resistance, but a target for novel bioreductive drugs?

**DOI:** 10.1038/bjc.1989.364

**Published:** 1989-11

**Authors:** P. Workman, M. I. Walton, G. Powis, J. J. Schlager

**Affiliations:** MRC Clinical Oncology Unit, Cambridge, UK.


Br. J. Cancer (1989), 60, 800-802 © The Macmillan Press Ltd., 1989

LETTER TO THE EDITOR

### DT-Diaphorase: questionable role in mitomycin C resistance, but a target for novel bioreductive drugs?

**Sir** – Marshall *et al.* (1989) reported recently that a human fibroblast cell line from a member of a cancer-prone family (3437T) was six times more resistant than an equivalent cell line from a normal donor (GM38) to the bioreductive antitumour antibiotic mitomycin C. Interestingly, this resistance was seen only when the drug exposure was carried out under well oxygenated conditions, but not under hypoxic conditions ($<10$ p.p.m. $O_2$). Similarly, a Chinese hamster ovary cell line with induced resistance to mitomycin C under oxic conditions did not exhibit comparable resistance under hypoxic conditions (Hoban *et al.*, 1989).

Compared to the normal human fibroblast line GM38, Marshall *et al.* (1989) reported that the resistant 3437T cell line exhibited substantially reduced levels of the enzyme DT-diaphorase or NAD(P)H: (quinone-acceptor) oxidoreductase (E.C.1.6.99.2) (Ernster *et al.*, 1987). Also, the DT-diaphorase inhibitor dicoumarol (3,3′-methylene-*bis* [4-hydroxycoumarin]) was shown to decrease the aerobic sensitivity of the normal GM38 line but not the resistant 3437T line. The conclusion was drawn that DT-diaphorase may play an important role in the bioreductive activation of mitomycin C under oxic conditions, and that deficient expression of this enzyme by the 3437T cell line leads (at least in part) to aerobic resistance to the drug. Similarly, this resistance is mimicked to some extent in the normal GM38 line by dicoumarol inhibition of the enzyme.

A number of studies on the mechanism of action of mitomycin C have relied heavily on the use of dicoumarol as a specific inhibitor of DT-diaphorase to probe for the functional role of this enzyme (e.g. Keyes *et al.*, 1984, 1985a, b; Dulhanty *et al.*, 1989). In one series of studies, the intriguing result was obtained that dicoumarol was able to increase the sensitivity of EMT6 mouse mammary tumour cells to mitomycin C under hypoxic conditions, yet decreased the sensitivity of the same cells when exposed to the drug under oxic conditions (Keyes *et al.*, 1984, 1985a, b). The increased toxicity under hypoxic conditions was related to the stimulation by dicoumarol of the amount of alkylating species generated (Keyes *et al.*, 1984). The clear and reasonable implication at the time the studies were conducted was that DT-diaphorase activates mitomycin C in air but serves to detoxify the same drug under hypoxia.

The proposed role for DT-diaphorase in the toxification of mitomycin C under aerobic conditions is contradictory to that which is generally thought to apply for simple quinones such as menadione (Lind *et al.*, 1982; Thor *et al.*, 1982; Morrison *et al.*, 1984; Ernster *et al.*, 1987). The aerobic toxicity of such compounds arises via one-electron reduction to the semiquinone free radical by enzymes such as NADPH:cytochrome P-450 reductase, leading to production of toxic oxygen species by auto-oxidation in a futile cycle (Figure 1). These radicals cause DNA and membrane damage. Since DT-diaphorase is an obligatory two-electron donor (Iyanagi & Yamazaki, 1970), reduction of quinones by this enzyme bypasses the toxic semiquinone radical by direct formation of the relatively stable hydroquinone, which can then undergo conjugation (Figure 1). Thus under aerobic conditions DT-diaphorase plays an important role in the cellular defense against oxygen stress caused by simple quinones, and inhibition by dicoumarol gives rise to increased toxicity (see Ernster *et al.*, 1987).

The picture is of course more complex with mitomycin C since toxicity under oxic conditions will arise not only via oxidative stress (Bachur *et al.*, 1979; Pritsos & Sartorelli, 1986) but also through bioreductive activation to DNA-alkylating species (Pan *et al.*, 1984; Tomasz *et al.*, 1987). Under hypoxic conditions DNA adducts, including cross-links, will predominate. It has been argued that both one-electron and two-electron reduction will result in bioreductive alkylation (Tomasz *et al.*, 1987; Hoey *et al.*, 1988). Nevertheless, the precise role of these two reduction mechanisms in this toxic pathway remains unclear.

Further complications to the ongoing controversy are suggested by correlations between DT-diaphorase activity and cytotoxicity obtained in two other recent studies. Pritsos *et al.* (1987) compared DT-diaphorase in three xenograft tumours grown in nude mice (human, equine and canine neoplasms) and found the lowest enzyme activity in the tumour with the greatest mitomycin C sensitivity. In addition, *in vivo* treatment of the sensitive tumour with mitomycin C resulted in a higher level of enzyme activity in the subsequently regrowing tumours. These correlations are consistent with a detoxifying function for DT-diaphorase *in vivo*. On the other hand, in the previously mentioned study of a Chinese hamster ovary cell line with induced resistance through exposure to mitomycin C under aerobic conditions *in vitro*, there was no measureable DT-diaphorase activity but a decrease in NADPH: cytochrome P-450 reductase (Hoban *et al.*, 1989; Walton *et al.*, 1989). These results indicate a more predominant role for cytochrome P-450 reductase in governing mitomycin C sensitivity. This enzyme is known to metabolise mitomycin C (Bachus *et al.*, 1979).

Much of the evidence in the continuing debate of the role of DT-diaphorase in mitomycin C bioactivation comes from studies such as those discussed earlier utilising dicoumarol as an inhibitor of the enzyme. There has, however, always been some concern regarding the over-reliance on dicoumarol as a

**Figure 1** Predominant roles of enzymatic one-electron versus two-electron transfer in the toxification versus detoxification reactions of simple quinones such as menadione. According to this scheme one-electron transferring enzymes such as NADPH: cytochrome P-450 reductase catalyse the formation of the semiquinone free radical, leading to the generation of the toxic species superoxide in the presence of oxygen. This pathway is bypassed as a result of direct two-electron reduction via DT-diaphorase, forming the more stable hydroquinone which can be further detoxified via conversion by conjugating enzymes to water soluble glucuronides and sulphates for excretion.

putative inhibitor of mitomycin C metabolism by DT-diaphorase where the measured end-point is cytotoxicity and not modulation of bioreductive metabolism. The potential for dicoumarol to modify cytotoxicity via additional mechanisms is clear (Keyes *et al.*, 1987; Akman *et al.*, 1985). In particular, dicoumarol was shown to increase hypoxic mitomycin C toxicity (but not reduce oxic toxicity) in L1210 cells with measurable DT-diaphorase activity (Keyes *et al.*, 1987). Potentiation of menadione toxicity in L1210 cells by a method not involving DT-diaphorase has also been proposed (Akman *et al.*, 1985). Indeed the authors of the various papers cited above, including Marshall *et al.* (1989), have been appropriately cautious in pointing out the potential artefacts of this approach.

In view of the contentious role of DT-diaphorase in mitomycin C toxicity, it is surprising that until recently there has been no published attempt to demonstrate metabolism of the drug by the purified enzyme. Such studies have now been carried out with several enzyme preparations and the results directly contradict a role for the enzyme in modulating mitomycin C sensitivity and resistance. The drug acts not as a substrate but in fact as an inhibitor of DT-diaphorase purified from human kidney by affinity and hydroxyapatite chromatography. This was demonstrated initially by two of us (Schlager & Powis, 1988) and confirmed independently by the others (Walton & Workman, unpublished). Mitomycin C is also not a substrate for DT-diaphorase purifed from rat liver (Powis & Schlager, unpublished). In addition, no dicoumarol-inhibitable metabolism of mitomycin C could be identified in DT-diaphorase-rich preparations obtained from the Chester Beatty strain of the rat Walker tumour (see later) or the HT29 human colon carcinoma (Walton & Workman, unpublished). In all cases the most direct assay involved analysis of mitomycin C using a sensitive and specific high-performance liquid chromotography assay, and we can now define the lower limit of measurable activity as $<30\,\mathrm{pmol\,min^{-1}\,Unit^{-1}}$ enzyme at $50\,\mu\mathrm{M}$ mitomycin C (where 1 Unit = 1 $\mu$mol cytochrome *c* reduced per min). As a positive control, we confirmed the observation of the reduction of 2,5-diaziridinyl-3,6-*bis*-(carboethoxyamino)-1,4-benzoquinone (AZQ) by HT29 cells (Siegel *et al.*, 1989) and also by Walker cells. Analogous to these findings was the demonstration that another complex quinone antitumour antibiotic, doxorubicin, was likewise not a substrate for rat liver DT-diaphorase (Wallin, 1986). On the other hand, Pritsos *et al.* (1987) claimed that mitomycin C was a substrate for partially purified beef liver DT-diaphorase with a $K_m$ of $1.5\,\mu\mathrm{M}$, although the assay method and detailed data were not presented.

DT-diaphorase exists in multiple forms (Ernster *et al.*, 1987) and it is conceivable that some forms but not others are able to bioactivate or detoxify mitomycin C. It is also possible that mitomycin C is metabolised by DT-diaphorase at a rate below the lower limit of analytical detection, but sufficient for biological activity. However, in view of the apparent inability of the human kidney, HT29 colon carcinoma and rat Walker tumour enzymes to metabolise mitomycin C as well as the potential for dicoumarol to exhibit pleiotropic pharmacological effects, we believe that evidence of dicoumarol modulation of cytotoxicity alone should no longer be accepted as evidence for the participation of DT-diaphorase in the bioactivation of this drug. Direct metabolic and enzymological data should be sought to complement such cytoxicity studies, so that the true role, if any, of DT-diaphorase in the metabolism of mitomycin C and related quinones can be definitively identified.

Should DT-diaphorase from a variety of sources fail to metabolise mitomycin C, it will be important to establish whether altered expression or activity of this enzyme is involved in other ways in the control of mitomycin C toxicity, or whether this is an epiphenomenon. It is interesting to note that rodent liver DT-diaphorase expression can be upregulated by two classes of xenobiotic inducers: (1) those such as polycyclic aromatics acting via the Ah locus and causing a simultaneous increase in phase 1 enzymes including cytochrome $P_1$-450; and (2) an alternative group including *tert*-butylhydroquinone and redox-labile diphenols capable of inducing phase 2 enzymes, such as UDPG-glucuronyl transferases, which normally play a detoxication role (see De Long *et al.*, 1987). DT-diaphorase has been shown to exhibit co-ordinately increased level of mRNA expression with glutathione-S-transferase Ya and Yb genes in rat hepatic preneoplastic nodules induced during chemical carcinogenesis in the Solt-Farber model, apparently as a result of hypomethylation of the gene (see Pickett, 1987).

Exciting new possibilities have been revealed by the recognition of close similarities in the biochemical profiles of rat preneoplastic and neoplastic hepatocytes and of *in vitro* derived multidrug resistant cells (Moscow & Cowan, 1988; Burt & Thorgeirsson, 1988). In both situations resistance to a range of toxins is seen; toxin accumulation is reduced; expression of the P-170 drug efflux membrane glycoprotein increases; protective phase 2 and related enzyme activities rise; and phase 1 enzymes may fall or rise. Thus expression of these various genes may depend on overlapping regulatory elements.

The role of DT-diaphorase in multidrug resistance is unknown. No up-regulation was observed in a multidrug-resistant MCF-7 human breast cancer line showing increased expression of glutathione-S-transferase $\pi$ and decreased aryl hydrocarbon hydroxylase activity; in fact a decrease in DT-diaphorase was seen (Vickers *et al.*, 1989). However, protein changes, including cytochrome P-450s, glutathione-S-transferases and even P-glycoprotein, are by no means consistent across all multidrug-resistant cell lines (neither is the cross-resistance profile), and a range of lines should now be examined to clarify this situation.

Increased expression of DT-diaphorase in tumour cells, including human breast and colon tumours, may be quite widespread (Koudstaal *et al.*, 1975; Schor & Cornelisse, 1983; Schor, 1987; Schlager & Powis, 1987, 1988). In view of these general findings, the presence of high DT-diaphorase levels in the HT29 human colon carcinoma and Walker 256 tumour cell lines makes these tumours especially appropriate models. It is interesting to note that the Chester Beatty strain of the Walker 256 rat tumour, though now an undifferentiated carcinosarcoma, originally arose as a mammary carcinoma of typical adenomatous structure (Earle, 1935; Rosenoer *et al.*, 1966).

Despite the apparent inability to metabolise mitomycin C, DT-diaphorase may represent an important target for drug bioactivation. In support of this, the elegant recent work of Knox *et al.* (1988*a*, *b*) has shown that high expression of DT-diaphorase in the Chester Beatty Walker 256 rat accounts for the extreme sensitivity of this tumour to CB 1954 (5-[aziridin-1-yl]-2,4-dinitrobenz-1-amide). DT-diaphorase reduces this agent in air to a highly toxic 4-hydroxylamine derivative. Moreover, we have recently shown similar activity for purified human kidney DT-diaphorase, and the Walker enzyme to have the ability to reduce the novel benzotriazine di-*N*-oxide hypoxic cell cytotoxin SR 4233 (3-amino-1,2,4-benzotriazine-1,4-dioxide) (unpublished data).

In summary, DT-diaphorase appears to play a questionable role in mitomycin C resistance, and further work is required to resolve this issue. DT-diaphorase may, however, provide an attractive target for the design of novel bioreductive drugs for the treatment of human tumours shown specifically to express high levels or an unusual form of this intriguing enzyme.

P. Workman and M.I. Walton,
MRC Clinical Oncology Unit,
Hills Road, Cambridge CB2 2QH, UK
and
G. Powis and J.J. Schlager,
Department of Pharmacology,
Mayo Clinic and Foundation,
200 First Street, SW,
Rochester, Minnesota 55905, USA.

We thank one of the referees for bringing to our attention the paper by Pritsos et al (1987), and a second referee together with Leon Cobb and Bernard Mitchley for information on the origin of the Walker 256 tumour.

The collaborative work between P.W., M.I.W., G.P. and J.J.S. was carried out under the auspices of the EORTC Pharmacokinetics and Metabolism Group.

### References

AKMAN, S.A., DIETRICH, M., CHLEBOWSKI, R., DOROSHOW, J. & BLOCK, J.B. (1985). Menadione (K3) and dicumarol (D) synergy *vs* leukaemia L1210. *Proc. Am. Assoc. Cancer Res.*, **26**, 325.

BACHUR, N.R., GORDON, S.L., GEE, M.V. & KON, H. (1979). NADPH-cytochrome P-450 reductase activation of quinone anticancer agents to free radicals. *Proc. Natl. Acad. Sci. USA*, **76**, 954.

BURT, R.K. & THORGEIRSSON, S.S. (1988). Coinduction of MDR-1 multidrug-resistance and cytochrome P-450 genes in rat liver by xenobiotics. *J. Natl. Cancer Inst.*, **80**, 1383.

DE LONG, M.J., SANTAMARIA, A.B. & TALALAY, P. (1987). Role of cytochrome $P_1$-P450 in the induction of NAD(P)H: quinone reductase in a murine hepatoma cell line and its mutants. *Carcinogenesis*, **8**, 1549.

DULHANTY, A.M., LI, M. & WHITMORE, G.F. (1989). Isolation of Chinese hamster ovary cell mutants deficient in excision repair and mitomycin C bioactivation. *Cancer Res.*, **49**, 117.

EARL, W.R. (1935). A study of the Walker rat mammary carcinoma 256, *in vivo* and *in vitro*. *Am. J. Cancer*, **24**, 566.

ERNSTER, L., ESTABROOK, R.W., HOCHSTEIN, P. & ORRENIUS, S. (eds) (1987). DT-diaphorase: a quinone reductase with special functions in cell metabolism and detoxication. *Chim. Scripta*, **27A.**

HOBAN, P.R., WALTON, M.I., ROBSON, C.N. & 5 others (1989). Mitomycin C resistance under aerobic but not hypoxic conditions in a mammalian cell line: Association with impaired drug activation and decreased NADPH: cytochrome P-450 reductive activity. *Cancer Res.*

HOEY, B.M., BUTLER, J. & SWALLOW, A.J. (1988). Reductive activation of mitomycin C. *Biochemistry*, **27**, 2608.

IYANAGI, T. & YAMAZAKI, I. (1970). One-electron transfer reactions in biochemical systems. V. Difference in the mechanism of quinone reduction by the NADH dehydrogenase and the NAD(P)H dehydrogenase (DT-diaphorase). *Biochim. Biophys. Acta*, **216**, 282.

KEYES, S.R., FRACASSO, P.M., HEIMBROOK, D.C., ROCKWELL, S., SLIGAR, S.G. & SARTORELLI, A.C. (1984). Role of cytochrome c reductase and DT-diaphorase in the biotransformation of mitomycin C. *Cancer Res.*, **44**, 5628.

KEYES, S.R., ROCKWELL, S. & SARTORELLI, A.C. (1985*a*). Enhancement of mitomycin C cytotoxicity to hypoxic cells by dicoumarol *in vivo* and *in vitro*. *Cancer Res.*, **45**, 213.

KEYES, S.R., ROCKWELL, S. & SARTORELLI, A.C. (1985*b*). Porfiromycin as a bioreductive alkylating agent with selective toxicity to hypoxic EMT6 tumor cells *in vivo* and *in vitro*. *Cancer Res.*, **45**, 3642.

KEYES, S.R., ROCKWELL, S. & SARTORELLI, A.C. (1987). Studies on the modulation of mitomycin C cytotoxicity by dicumarol. *Proc. Am. Assoc. Cancer Res.*, **28**, 411.

KNOX, R.J., BOLAND, M.P., FRIEDLOS, F., COLES, B., SOUTHAN, C. & ROBERTS, J.J. (1988*a*). The nitroreductase in Walker cells that activates 5-(aziridin-1-yl)-2,4-dinitrobenzamide (CB 1954) to 5-aziridin-1-yl)-4-hydroxylamino-2-nitrobenzamide is a form of NAD(P)H dehydrogenase (quinone) (EC 1.6.99.2). *Biochem. Pharmacol.*, **37**, 4671.

KNOX, R.J., FRIEDLOS, F., JARMAN, M. & ROBERTS, J.J. (1988*b*). A new cytotoxic, DNA interstrand crosslinking agent, 5(aziridin-1-yl)-4-hydroxylamino-2-nitrobenzamide, is formed from 5-(aziridin-1-yl)-2,4-dinitrobenzamide (CB 1954) by a nitroreductase enzyme in Walker carcinoma cells. *Biochem. Pharmacol.*, **37**, 4661.

LIND, C., HOCHSTEIN, P. & ERNSTER, L. (1982). DT-diaphorase as a quinone reductase: a cellular device against semiquinone and superoxide formation. *Arch. Biochem. Biophys.*, **216**, 178.

MARSHALL, R.S. & RAUTH, A.M. (1989). Deficient activation by a human cell strain leads to mitomycin resistance under aerobic but not hypoxic conditions. *Br. J. Cancer*, **59**, 341.

MORRISON, H., JERNSTROM, B., NORDENSKJOLD, M., THOR, H. & ORRENIUS, S. (1984). Induction of DNA damage by menadione (2-methyl-1,4-napthaquinone) in primary cultures of rat hepatocytes. *Biochem. Pharmacol.*, **33**, 1763.

MOSCOW, J.A. & COWAN, K.H. (1988). Multidrug resistance. *J. Natl Cancer Inst.*, **80**, 14.

PAN, S.S., ANDREWS, P.A. & GLOVER, C.J. (1984). Reductive activation of mitomycin C and mitomycin C metabolites catalyzed by NADPH-cytochrome P-450 reductase and xanthine oxidase. *J. Biol. Chem.*, **259**, 959.

PICKETT, C.B. (1987). Structure and regulation of glutathione S-transferase genes. *Essays Biochem.*, **23**, 116.

PRITSOS, C.A., PARDINI, L.L., ELLIOT, A.J. & PARDINI, R.S. (1987). Relationship between the antioxidant enzyme DT-diaphorase and tumour response to mitomycin C treatment. In *Oxygen Radicals in Biology and Medicine*, Simic, M.G. & Taylor, K.A. (eds) p. 713. Plenum Press: New York.

PRITSOS, C.A. & SARTORELLI, A.C. (1986). Generation of reactive oxygen radicals through bioactivation of mitomycin C antibiotics. *Cancer Res.*, **46**, 3528.

ROSENOER, V.M., MITCHLEY, B.C.V., ROE, F.J.C. & CONNORS, T.A. (1986). Walker carcinosarcoma 256 in a study of anticancer agents. I. Method for simultaneous assessment of therapeutic value and toxicity. *Cancer Res.*, **26**, suppl. 2, 937.

SCHLAGER, J.J. & POWIS, G. (1987). NAD(P)H:(quinone-acceptor) oxidoreductase (QAO, E.C.1.6.99.2) activity in human normal and tumour tissues. *Pharmacologist*, **29**, 177.

SCHLAGER, J.J. & POWIS, G. (1988). The effect of smoking on human cytosolic DT-diaphorase (DT) (E.C.1.6.99.2) activity in normal and tumour tissues. *Proc. Am. Assoc. Cancer Res.*, **29**, 8.

SCHOR, N.A. (1987). DT-diaphorase and the cancer cell. *Chim. Scripta*, **27A**, 135.

SIEGEL, D., PACHECO, D.Y., GIBSON, N.W. & ROSS, D. (1989). Mechanisms of cytotoxicity associated with the two electron reduction of 2,5-diaziridinyl-3,6-bis (carboethoxyamino)-1,4-benzoquinone (AZQ) in human colon carcinoma cells. *Proc. Am. Assoc. Cancer Res.*, **30**, 558.

THOR, H., SMITH, M.T., HARTZELL, P., BELLAMO, G., JEWELL, S.A. & ORRENIUS, S. (1982). The metabolism of menadione by isolated hepatocytes. *J. Biol. Chem.*, **257**, 12419.

TOMASZ, M., LIPMAN, R., DONDAPATI, C., PAWLAK, J., VERDINE, G.L. & NAKANISHI, K. (1987). Isolation and structure of a covalent cross-link adduct between mitomycin C and DNA. *Science*, **235**, 1204.

VICKERS, P.J., TOWNSEND, A.J. & COWAN, K.H. (1989). Mechanisms of resistance to antineoplastic drugs. In *Developments in Cancer Chemotherapy*, Glazer, R.I. (ed) p. 117. CRC Press: Boca Raton.

WALTON, M.I., HOBAN, P.R., ROBSON, C.N., WORKMAN, P., HARRIS, A.L. & HICKSON, I.D. (1989). Mitomycin C resistance: association with decreased NADPH cytochrome P-450 reductase activity in Chinese hamster ovary (CHO) cells *in vitro*. *Br. J. Cancer* (in the press).

WALLIN, R. (1986). Adriamycin and DT-diaphorase. *Cancer Lett.*, **30**, 97.