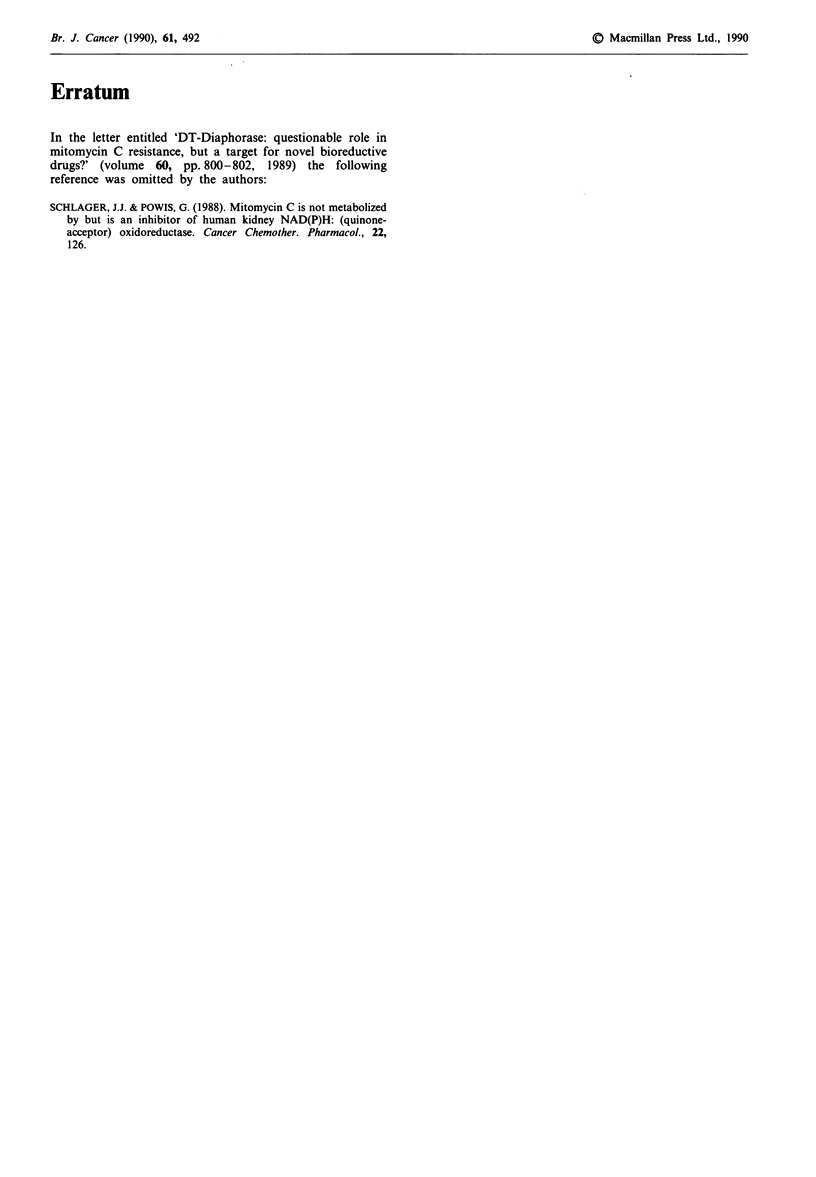# Erratum

**Published:** 1990-03

**Authors:** 


 © Macmillan Press Ltd., 1990

### Erratum

In the letter entitled 'DT-Diaphorase: questionable role in mitomycin C resistance, but a target for novel bioreductive drugs?' (volume **60**, pp. 800–802, 1989) the following reference was omitted by the authors:

SCHLAGER, J.J. & POWIS, G. (1988). Mitomycin C is not metabolized by but is an inhibitor of human kidney NAD(P)H: (quinone-acceptor) oxidoreductase. *Cancer Chemother. Pharmacol.*, **22**, 126.